# NK cell expression of natural cytotoxicity receptors may determine relapse risk in older AML patients undergoing immunotherapy for remission maintenance

**DOI:** 10.18632/oncotarget.5559

**Published:** 2015-10-30

**Authors:** Anna Martner, Anna Rydström, Rebecca E. Riise, Johan Aurelius, Mats Brune, Robin Foà, Kristoffer Hellstrand, Fredrik B. Thorén

**Affiliations:** ^1^ TIMM Laboratory, Sahlgrenska Cancer Center, University of Gothenburg, Gothenburg 405 30, Sweden; ^2^ Department of Hematology, University of Gothenburg, Gothenburg 413 45, Sweden; ^3^ Department of Cellular Biotechnologies and Hematology, Sapienza University of Rome, Rome 00161, Italy

**Keywords:** acute myeloid leukemia, immunotherapy, natural killer cells, NKp30, NKp46

## Abstract

In a phase IV trial, eighty-four patients (age 18–79) with acute myeloid leukemia (AML) in first complete remission (CR) received cycles of immunotherapy with histamine dihydrochloride (HDC) and low-dose human recombinant interleukin-2 (IL-2) to prevent relapse in the post-consolidation phase. Aspects of natural killer (NK) cell biology were analyzed before and during immunotherapy with focus on outcome in older patients. In younger (<60 years old, *n* = 37) and older patients (>60 years old, *n* = 47), treatment with HDC/IL-2 resulted in an expansion of CD56^bright^ and CD16^+^ NK cells in blood along with an increased NK cell expression of the natural cytotoxicity receptors (NCR) NKp30 and NKp46. In older patients, a high expression of NKp30 or NKp46 on CD16^+^ NK cells before and during therapy predicted leukemia-free and overall survival. These results suggest that NK cell functions determine relapse risk and survival in older AML patients and point to biomarkers of efficacy in protocols for remission maintenance.

## INTRODUCTION

At diagnosis, approximately 70% of patients with acute myeloid leukemia (AML) are >60 years old. While a high proportion of older patients achieve complete remission (CR) after chemotherapy, leukemia relapse is common in the post-chemotherapy phase and significantly explains why the rates of 5-year survival of older patients are in the range of 10–15% [1–4]. Immunotherapy with histamine dihydrochloride and low-dose interleukin-2 (HDC/IL-2) aims at boosting anti-leukemic functions of natural killer (NK) cells to reduce or eradicate residual leukemia [5–7]. In a phase III trial, treatment with HDC/IL-2 was shown to prevent relapse in AML patients in CR [8].

Several aspects of NK cell function are reportedly relevant to AML prognosis [7, 9–12], but a systematic analysis of aspects of NK cell biology in older patients has not been carried out. Human NK cells comprise two main phenotypes: the cytotoxic CD16^+^/56^+^ NK cells (here referred to as CD16^+^ NK cells) constitute 90–95% of blood NK cells in healthy subjects, whereas the weakly cytotoxic CD16^−^/56^bright^ cells (CD56^bright^ NK cells) are regarded as precursors of CD16^+^ NK cells [13, 14]. NK cell cytotoxicity is regulated by activating and inhibitory NK cell receptors and their cognate ligands on malignant target cells. The main activating receptors comprise the natural cytotoxicity receptors (NCRs; NKp46, NKp30 and NKp44) and NKG2D [15–18]. We have previously published an interim report of aspects of NK cell biology in AML patients who participated in a phase IV trial using HDC/IL-2 for remission maintenance [19]. Here, we report final results from this trial, with a focus on the subgroup of older AML patients (>60 years).

## RESULTS AND DISCUSSION

**Figure 1:**
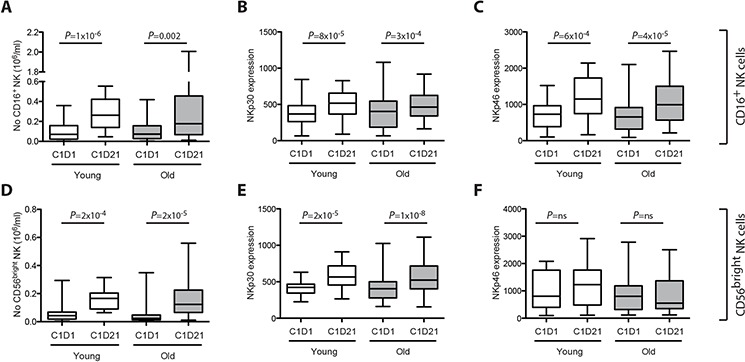
Induction and activation of NK cells in patients below and above 60 years of age The box plots in panel A-F show the number of CD16^+^ or CD56^bright^ NK cells in blood **A and D.** and the median fluorescence intensity (MFI) of NKp30 **B and E.** and NKp46 **C and F.** on CD16^+^ or CD56^bright^ NK cells before (C1D1) and after (C1D21) the first 21-day treatment cycle with HDC/IL-2 in patients <60 years (young, white; NK cell counts, *n* = 17, NCR expression, *n* = 21) and >60 years (old, grey; NK cell counts, *n* = 30, NCR expression, *n* = 35). Induction of NK cell counts and NCR expression were analyzed using Student's paired *t*-test.

AML patients in first CR received ten three-week cycles of HDC/IL-2 over 18 months as described elsewhere [8]. Blood samples were drawn before and after the first cycle for analysis of absolute NK cell counts and NK cell expression of NCRs. A previous interim report [19] showed that treatment with HDC/IL-2 augmented NK cell counts in the blood along with an increased NK cell expression of NKp30 and NKp46 during treatment cycles. We first compared the degree of NK cell and NCR induction in younger (<60 years) and older (>60 years) patients, respectively. The NK cell counts and NCR expression at onset of treatment did not differ between these age groups with the exception that the ratio of CD56^bright^ to CD16^+^ cells was lower in older patients (*P* = 0.045, Mann-Whitney Test), which is in agreement with previous findings in healthy subjects [20]. Younger and older patients did not differ regarding the accumulation of CD16^+^ and CD56^bright^ NK cells in the blood during treatment cycles (Fig. 1A, 1D) or induction of NK cell expression of NKp30 and NKp46 receptors (Fig. 1B–1C, 1E–1F).

**Figure 2:**
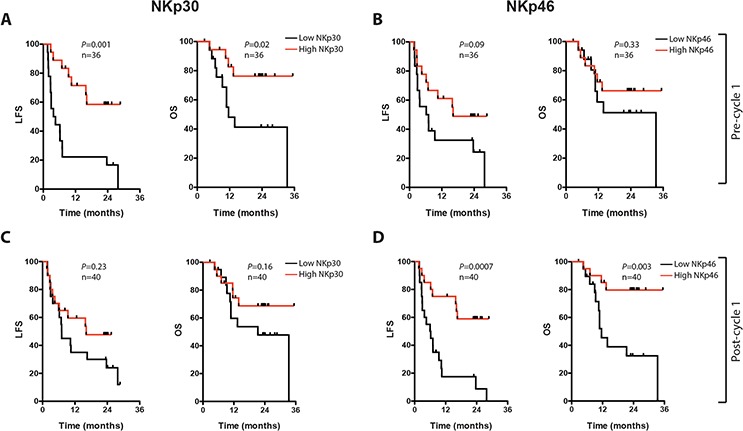
Impact of NK cell NCR expression on leukemia-free survival (LFS) and overall survival (OS) in older AML patients In panels **A-D**, LFS and OS are shown for older patients (>60), dichotomized based on above (red) or below (black) median of NKp30 **A and C.** or NKp46 **B and D.** expression on CD16^+^ NK cells before (A and B) or after (C and D) the first HDC/IL-2 treatment cycle.

We aimed at determining the impact of NK cell-related markers on outcome by dichotomizing younger and older patients by high or low (by the median) NK cell counts or NK cell NCR expression intensity followed by analysis of leukemia-free survival (LFS) and overall survival (OS). NK cell counts did not significantly predict outcome in younger or older patients, and NK cell NCR expression did not predict outcome in younger patients (*P* > 0.5 for LFS, not shown). Older patients with an above-median expression of NKp30 on CD16^+^ NK cells at the onset of therapy (cycle 1 day 1; C1D1) showed improved LFS and OS, with a similar trend for NKp46 (Fig. 2A and 2C). A high expression of NKp46 on CD16^+^ NK cells after the first treatment cycle (cycle 1 day 21; C1D21) was positively associated with LFS and OS with a similar trend for NKp30 (Fig. 2B and 2D). No significant associations were observed between outcome and the level of induction of NK cell counts or the induction of NCRs during the first treatment cycle (i.e. C1D21 minus C1D1 levels, not shown). In multivariate analyses corrected for age, risk group classification, number of induction courses required to achieve CR and number of consolidation courses, NKp30 expression on C1D1 and NKp46 expression on C1D21 independently predicted LFS and/or OS in older patients (Table 1). One patient in CR died of sepsis whereas all other deaths were preceded by a relapse, which illustrates the impact of relapse for survival in the post-consolidation phase [21] also in the group of elderly AML patients.

**Table 1:** Univariate and multivariate analyses of the impact of NKp30 or NKp46 expression on LFS and OS

Variable	Univariate analysis			Multivariate analysis		
	Hazard ratio	Confidence interval	*p*-value	Hazard ratio	Confidence interval	*p*-value
NKp30, C1D1, LFS	0.24	0.099–0.61	0.002	0.14	0.043–0.43	0.001
NKp30, C1D1, OS	0.28	0.088–0.91	0.034	0.17	0.037–0.78	0.022
NKp46, C1D21, LFS	0.25	0.10–0.59	0.001	0.28	0.093–0.82	0.020
NKp46, C1D21, OS	0.21	0.067–0.66	0.007	0.22	0.047–1.05	0.058

Univariate and multivariate Cox regression analyses were utilized to determine the impact of above or below median expression levels of NKp30 cycle 1 day 1 (C1D1) or NKp46 cycle 1 day 21 (C1D21) on LFS and OS in patients >60 years old. In the multivariate analyses, hazard ratios were corrected for age, risk group classification, number of induction courses needed to achieve CR, and number of consolidation courses

The reason for the lack of correlation between NCR expression and outcome in younger patients is not known, but may relate to the lower number of younger patients in the study, along with a lower incidence of relapse and death in this age group (Supplementary Figure S1). NK cell function relies on interactions between NCR and their ligands on target cells with ensuing activation of NK cell cytotoxicity [22]. While NCR ligands are frequently expressed by malignant AML cells [9], NK cells of newly diagnosed AML patients may express lower densities of NCR [11], which impacts on the anti-leukemic efficiency of NK cells as well as on survival and the likelihood of achieving CR after chemotherapy [23]. Our results extend these previous findings by pointing to a role for NCR in preventing relapse in the post-chemotherapy phase of AML. Further studies are required to confirm these results and to establish whether NK cell NCR expression heralds relapse also in untreated patients or in patients undergoing other immunotherapies.

## MATERIALS AND METHODS

### Patients

This single-armed multicenter phase IV study (Re:Mission, NCT01347996, registered at www.clinicaltrials.gov) enrolled 84 patients (age 18–79) with AML in first CR who received ten 21-day cycles of HDC/IL-2 for 18 months or until relapse or death. Primary endpoints included assessment of the quantitative and qualitative pharmacodynamic effects of HDC/IL-2 by monitoring NK cell phenotypes and their functionality before and after the first treatment cycle. The protocol stated that data collected in support of these objectives were to be analyzed for the defined populations as a whole and by subgroups according to patient age at enrollment (<60 and > 60 years). The Analyses of NK cell counts and NCR expression vs. outcome were performed post-hoc. The characteristics at enrollment of older patients (>60 years, median 67.2, range 60–79) are accounted for in Table 2. Details of patient characteristics, induction and consolidation therapy, exclusion criteria, treatment and dosing are found in a previous interim report [19].

**Table 2:** Patient characteristics (age >60)

	*n* (%)	LFS (%)
**Sex**		
Female	23 (49)	6/23 (26)
Male	24 (51)	10/24 (42)
**Risk group**		
Favorable risk	17 (36)	8/17 (47)
Intermediate I	10 (21)	2/10 (20)
Intermediate II	9 (19)	4/9 (44)
High risk	6 (13)	1/6 (17)
ND	5 (11)	1/5 (20)
**Karyotype**		
Normal	23 (49)	9/23 (39)
Favorable	5 (11)	2/5 (40)
Unfavorable	6 (13)	2/6 (33)
Other	10 (21)	3/10 (30)
ND	3 (6)	0/3 (0)
**Mutation status**		
NPM1 (*n* = 39)	14 (36)	6/14 (43)
FLT3 (*n* = 37)	3 (8)	0/3 (0)
CEBPA (*n* = 23)	1 (4)	0/1 (0)
**Induction courses (*n* = 46)**		
1	32 (70)	14/32 (44)
>1	14 (30)	2/14 (14)
**Consolidation courses (*n* = 46)**		
0–2	26 (57)	6/26 (23)
> 2	20 (43)	10/20 (50)

### Sampling of peripheral blood and flow cytometry

Peripheral blood was collected before and after treatment cycle 1, i.e. on day 1 and day 21 of cycle 1 (C1D1 and C1D21), and PBMC were isolated and cryopreserved at local sites and shipped on dry ice to the central laboratory (at the Sahlgrenska Cancer Center, University of Gothenburg, Sweden) for flow cytometry analysis. PBMC samples were stained with fluorochrome-conjugated antibodies and a viability marker and analyzed using a 4-laser BD LSRFortessa SORP (BD Biosciences, San Diego, CA), as accounted for in detail elsewhere [19].

Samples were available from 32 out of 37 younger patients and from 45 out of 47 older patients. All available samples were analyzed. If an analysis failed according to pre-defined criteria (experimental failure, few cells, poor cellular viability), a second sample was thawed for re-analysis. In 18 cases for C1D1 samples and in 12 cases for C1D21 samples, also the second attempt failed to generate data, and these samples were excluded from analysis. Differential counts of whole blood were performed at local sites and were utilized to calculate absolute counts of blood NK cells. Differential counts were lacking from four younger and five older patients.

### Statistics

In accordance with the statistical plan, paired *t*-test was used for single comparisons of NK cell phenotypes. The analyses of NK cell markers vs. outcome are based on data for LFS, defined as the time in days from start of immunotherapy with HDC/IL-2 to relapse or death from any cause) and OS available at the trial closing date (October 13, 2014), i.e. when all patients had been followed for at least 24 months (18 months of treatment and 6 months of additional follow-up). Relapse was defined as at least 5% blast cells in the bone marrow or by the occurrence of extramedullary leukemia. LFS was defined as the time from the first day of treatment with HDC/IL-2 to relapse or death from any cause. OS was defined as the corresponding time to death regardless of cause. LFS and OS were analyzed using the log-rank test. Parameters that significantly predicted LFS and/or OS were further analyzed by univariate and multivariate Cox regression analysis. In the multivariate analyses, hazard ratios were corrected for age, risk group classification, number of induction courses required to achieve CR (1 or >1) and number of consolidation courses (0–2 or >2; Table 1). All indicated *P*-values are 2-sided. Patients were risk-classified according to recommendations by the European LeukemiaNet [24]. The trial was approved by the Ethics Committees of each participating institution, and all patients gave written informed consent before enrollment.

## SUPPLEMENTARY FIGURE


