# Supplementary material for: NK cell expression of natural cytotoxicity receptors may determine relapse risk in older AML patients undergoing immunotherapy for remission maintenance
Source: Oncotarget. 2015 Oct 30;6(40):42569–74. doi: 10.18632/oncotarget.5559 (PMC4767453; doi:10.18632/oncotarget.5559)
Supplement: Supplementary file 1 [file oncotarget-06-42569-s001.pdf]

SUPPLEMENTARY FIGURE

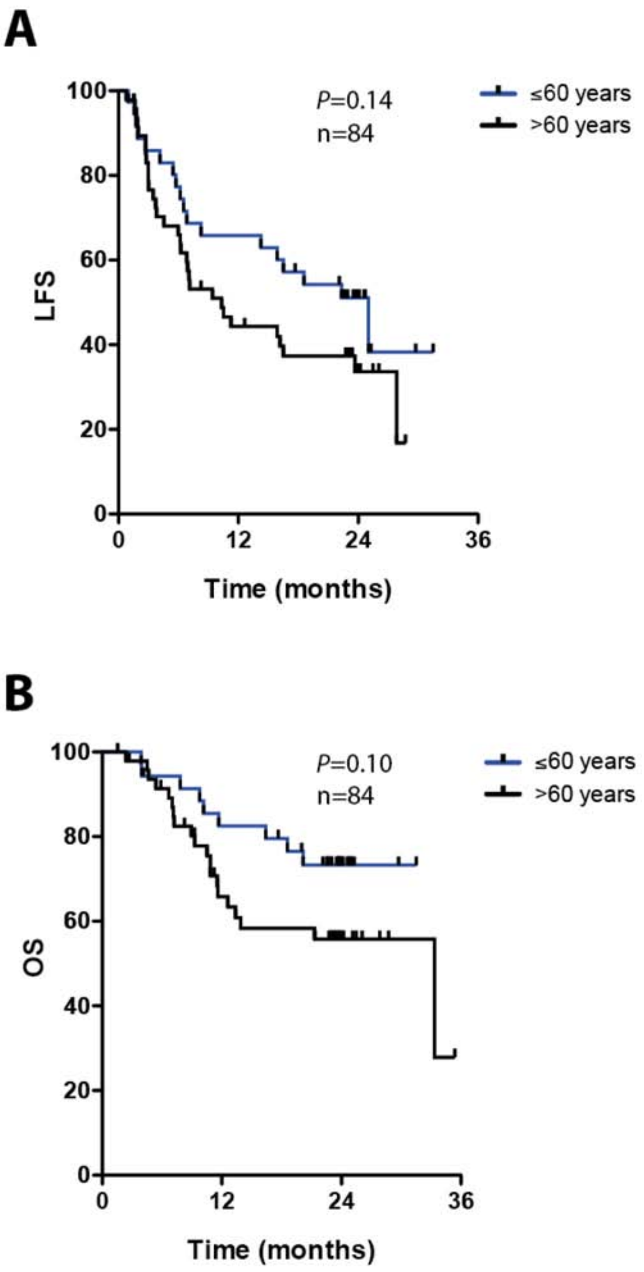

Supplementary Figure S1: LFS and OS in younger and older patients. **Panels A and B** show LFS and OS for older patients (>60 years old; black line) and younger patients (<60 years old; blue line).
